# Factors affecting the efficacy of small bowel capsule endoscopy in patients with chronic abdominal pain

**DOI:** 10.3389/fmed.2024.1515823

**Published:** 2025-02-20

**Authors:** Xinlong He, Qing Xu, Hanbing Xue

**Affiliations:** ^1^Division of Gastroenterology and Hepatology, NHC Key Laboratory of Digestive Diseases, Renji Hospital, Shanghai Jiao Tong University School of Medicine, Shanghai, China; ^2^Department of Gastrointestinal Surgery, Renji Hospital, Shanghai Jiao Tong University School of Medicine, Shanghai, China

**Keywords:** small bowel capsule endoscopy, chronic abdominal pain, delayed upper gastrointestinal transit, lesion detection rate, regression analysis

## Abstract

**Background:**

Abdominal pain is a common clinical symptom, and the role of small bowel capsule endoscopy (SBCE) in the evaluation of abdominal pain remains a subject of ongoing debate. The objective of this study is to investigate the factors influencing the efficacy of SBCE in patients with chronic abdominal pain.

**Methods:**

This study retrospectively analyzed the medical records of patients presenting with chronic abdominal pain as the primary complaint who underwent SBCE at Renji Hospital from January 2014 and January 2023. Data collection included patient demographics and relevant influencing factors, such as hospitalization status, anemia, elevated inflammatory markers, hypertension, and diabetes. Univariate and multivariate analyses were employed to examine the factors associated with SBCE transit status and positive outcomes.

**Results:**

A total of 524 patients were included in the study, of whom 113 presented with DUGT and received conservative observation, pharmacological treatment, or endoscopic intervention as appropriate. The overall completion rate was 97.1%. Hospitalization status, diabetes, and anemia were identified as risk factors for DUGT in multivariate analyses. Positive lesions were detected in 160 cases, yielding an overall lesion detection rate of 30.5%. Furthermore, multivariate regression analysis indicated that anemia (hemoglobin <90 g/L) and elevated inflammatory markers were associated with a higher rate of positivity.

**Conclusion:**

In conclusion, our study found that hospitalization status, diabetes, and anemia as significant risk factors for DUGT in patients with chronic abdominal pain. Furthermore, we found that SBCE is highly effective in detecting lesions in patients with chronic abdominal pain combined with anemia and elevated inflammatory markers.

## Introduction

Chronic abdominal pain is currently a challenging clinical problem in modern society. The term “chronic pain” is defined as persistent or recurrent pain lasting 3 months or longer (1, 2). Some studies (3, 4) have demonstrated that abdominal pain represents the most prevalent gastrointestinal symptom observed in clinical practice, with a prevalence rate ranging from 22 to 25%. Abdominal pain, as a symptomatic complaint can be caused by a number of disorders, including both gastrointestinal and extra-intestinal conditions that involve the genitourinary tract, abdominal wall, thorax, and spine. On the other side of the spectrum, it may be caused by organic diseases (Crohn’s disease, intestinal obstruction, chronic appendicitis, chronic pancreatitis, etc.) or functional disorders (functional dyspepsia, irritable bowel syndrome, and centrally mediated abdominal pain syndrome, etc.) (1). The complexity of these conditions necessitates a wide range of differential diagnostic tests and examinations. In some cases, despite extensive and costly investigations, the underlying cause of the abdominal pain may remain undetermined, placing a significant strain on healthcare systems worldwide.

Since its clinical introduction in the early 21st century, SBCE has proven valuable in diagnosing and managing small bowel-related conditions, including unexplained gastrointestinal bleeding, Crohn’s disease, and small bowel tumors (5). However, the effectiveness of this test in patients whose primary complaint is chronic abdominal pain remains a subject of debate. Generally, SBCE is not recommended as a first-line diagnostic tool for patients experiencing chronic abdominal pain. Bardan et al. (6) and Fry et al. (7) concluded that SBCE has no significant clinical value in the diagnosis of patients with chronic abdominal pain, but, May et al. (8) concluded that SBCE has a role in patients with chronic abdominal pain who have some additional symptoms or signs. Therefore, the aim of our study was to determine the factors affecting the efficacy of SBCE examination, thus enhancing the utilization of the capsule in patients with chronic abdominal pain, as well as reducing the waste of healthcare resources.

## Methods

### Patients and study design

We retrospectively included 524 patients who underwent SBCE at our institution from January 2014 to January 2023, all presenting with chronic abdominal pain as their chief complaint. None of these patients exhibited lesions that could explain their symptoms during routine clinical examinations and laboratory tests, which included blood tests, urinalysis, fecal tests, ultrasonography, computed tomography imaging, esophagogastroduodenoscopy, and colonoscopy. We collected demographic characteristics of the patients, including their names, genders, ages, heights, weights, underlying diseases, and specific details from the SBCE examinations, such as hemoglobin levels and inflammatory markers (white blood cell count, platelet count, C-reactive protein, erythrocyte sedimentation rate, and calcitoninogen). The study received approval from the Institutional Review Board of Renji Hospital.

In our study, chronic abdominal pain was defined as recurrent or persistent abdominal pain lasting more than 3 months. Anemia was defined as a hemoglobin level of less than 90 g/L. Small bowel lesions were categorized as clinically significant or not. Clinically significant lesions included mucosal breakdowns such as erosions, ulcers, strictures, or tumors. Lesions that were not considered clinically significant included arteriovenous malformations, red spots, and erythema, etc., and it is possible that these lesions did not account for the patients’ symptoms. The lesion detection rate was defined as the percentage of patients with positive small bowel lesions relative to the total number of patients examined. Delayed upper gastrointestinal transit (DUGT) was defined as the inability of a capsule to pass through the pylorus within 1 h.

After signing the informed consent form for the SBCE examination, all patients received the PillCam SB2/3. They were instructed to fast for 12 h prior to the examination and to adequately prepare their bowels. Approximately 2–3 h after swallowing the capsule, patients were permitted to consume clear liquids and resume their daily activities. At the conclusion of the examination, patients returned the recording system, and the physician downloaded the video images into a computerized system for analysis by a specialized endoscopist. For patients exhibiting signs of delayed transit, conservative treatment or endoscopic intervention to facilitate passage to the duodenum was chosen based on clinical experience.

### Statistical analysis

Statistical analysis was conducted using SPSS version 26.0. Count data were expressed as % and were compared using the *χ*^2^ test. Factors associated with DUGT were identified through logistic regression analysis. Odds ratios (OR) and their corresponding 95% confidence intervals (CI) were calculated. A *p*-value of less than 0.05 was considered statistically significant.

## Results

### Demographical and clinical characteristics

A total of 524 patients with chronic abdominal pain underwent SBCE, 345 males and 179 females, with a median age of 45 years. Of the cases examined, 113 were delayed, and all patients received conservative, pharmacologic, or endoscopic interventions (Table 1). A total of 509 patients completed the examination, resulting in an examination completion rate of 97.1%.

**Table 1:** Univariate analysis of factors influencing the findings and DUGT of SBCE.

		*n*	DUGT		*p*-value	Positive findings on SBCE		Detection rate (%)	*P*-value
			No (*n* = 411)	Yes (*n* = 113)		No (*n* = 364)	Yes (*n* = 160)		
Sex, *n*	Male	345	262	83	0.054	244	101	29.3%	0.385
	Female	179	149	30		120	59	33%	
Age (yr)	>60	97	76	21	0.982	69	28	28.9%	0.693
	≤60	427	335	92		295	132	30.9%	
BMI (kg/m^2^)	>23.9	156	118	38	0.311	114	42	26.9%	0.243
	≤23.9	368	293	75		250	118	32.1%	
Hospitalization	Yes	142	93	49	<0.001	89	53	37.3%	0.040
	No	382	318	64		275	107	28%	
Hypertension	Yes	23	19	4	0.812	15	8	34.8%	0.651
	No	501	392	109		349	152	30.3%	
Diabetes	Yes	31	12	19	<0.001	15	16	51.6%	0.009
	No	493	399	94		349	144	29.2%	
Hypokalemia	Yes	20	11	9	0.020	12	8	40%	0.349
	No	504	400	104		352	152	30.2%	
Diarrea	Yes	34	23	11	0.114	23	11	32.4%	0.812
	No	490	388	102		341	149	30.4%	
Anemia	Yes	23	7	16	<0.001	9	14	60.9%	0.001
	No	501	404	97		355	146	29.1%	
Elevated inflammatory biomarker	Yes	44	38	6	0.182	21	23	52.3%	0.001
	No	480	373	107		343	137	28.5%	
Small bowel transit time	≤4 h	152	/	/	/	111	41	27%	0.420
	>4 h	357	/	/	/	248	109	30.5%	
Gastric transit time	≤1 h	411	/	/	/	283	128	31.1%	0.564
	>1 h	113	/	/	/	81	32	28.3%	

### Risk factors for DUGT

Univariate analysis revealed that hospitalization status, diabetes, hypokalemia, and anemia were significantly associated with DUGT in patients presenting with chronic abdominal pain (Table 1). Further multivariate regression analysis indicated a significant association between hospitalization status (OR 1.686, 95% CI: 1.011–2.809, *p* = 0.045), diabetes (OR 3.463, 95% CI: 1.384–8.666, *p* = 0.008), and anemia (OR 4.916, 95% CI: 1.798–13.444, *p* = 0.002) as risk factors for DUGT (Table 2).

**Table 2:** Multivariate analysis of factors influencing the findings and DUGT of SBCE.

	*P*-value	OR (95% CI)
DUGT		
Hospitalization	0.045	1.686(1.011 2.809)
Diabetes	0.008	3.463(1.384 8.666)
Hypokalemia	0.547	0.691(0.207 2.306)
Anemia	0.002	4.916(1.798 13.444)
Positive findings on SBCE		
Diabetes	0.411	1.434(0.607 3.387)
Hospitalization	0.750	1.079(0.675 1.726)
Elevated inflammatory biomarker	0.019	2.218(1.141 4.315)
Anemia	0.047	2.622(1.011 6.798)

### Factors influencing the positive outcomes of SBCE

A total of 160 patients were identified with positive lesions, resulting in an overall lesion detection rate of 30.5%. The specific lesions were categorized as follows: polyps (22 cases), ulcers (108 cases), enterostenosis (10 cases), vascular lesions (17 cases), red spots (18 cases), mucosal congestion and edema (111cases), suspected submucosal lesions (21 cases), diverticula (16 cases), lymphatic dilatations (34 cases), erosions (43 cases), and others (38 cases) (Table 3). Univariate analysis revealed that diabetes, elevated inflammatory markers, and anemia were significantly associated with the lesion detection rate in patients presenting with abdominal pain (Table 1). Further multivariate regression analysis indicated that elevated inflammatory markers (OR 2.218, 95% CI: 1.141–4.315, *p* = 0.019) and anemia (OR 2.622, 95% CI: 1.011–6.798, *p* = 0.047) were significant factors influencing the lesion detection rate (Table 2).

**Table 3:** Findings of SBCE.

Findings of SBCE	
Lesions considered significant	
Ulcers	108
Erosions	43
Enterostenosis	10
Suspected submucosal lesions	21
Lesions considered not significant	
Polyps	22
Vascular lesions	17
Red spots	18
Mucosal congestion and edema	111
Diverticula	16
Lymphatic dilatations	34
Others^*^	38

*Include small bowel villous changes, hemorrhages, lymphoid follicular hyperplasia, mucosal scarring, etc.

## Discussion

Our study identified hospitalization status, diabetes, and anemia as risk factors for DUGT in patients experiencing chronic abdominal pain. Additionally, we found that elevated inflammatory markers and anemia were significantly associated with lesion detection rates. Notably, anemia was both a risk factor for DUGT and significantly correlated with a high lesion detection rate, which may raise concerns during the screening of patients with chronic abdominal pain. Statistically, we observed that the DUGT rate among patients with chronic abdominal pain and anemia was 69.6%. However, the examination completion rate reached 100% when appropriate measures were implemented, and the lesion detection rate approached 60.9%. Therefore, we believe it is worthwhile to conduct SBCE examinations for patients with chronic abdominal pain with anemia after comprehensive consideration.

A retrospective study conducted in Europe involving 190 patients who underwent SBCE found that persistent hospitalization status was linked to an increased risk of gastric retention. This condition occurred in 24.1% of patients during their hospitalization. The study also revealed that persistent hospitalization status was associated with lower rates of completion for whole small bowel examinations (9). In a prior prospective study in Japan (10), 76 patients undergoing SBCE were recruited to evaluate the relationship between physical activity and examination completion rates. The completion rate for SBCE was 100% in the outpatient group, 85.7% in the mild bed rest group, and 72.2% in the strict bed rest group. The investigators concluded that low physical activity was a significant risk factor for failure to reach the cecum during SBCE, and that completion rates increased with higher levels of patient physical activity. Additionally, our previous study with a large sample size also confirmed that hospitalization status was strongly associated with the completion rate of SBCE examinations (11). In our current study, we further confirmed that hospitalization status was a significant risk factor for DUGT in patients with chronic abdominal pain. We believed this was related to extended bed rest and activity limitations experienced by hospitalized patients. Therefore, based on these findings, endoscopists should closely monitor the status of capsule operations when hospitalized patients undergo SBCE examinations. If necessary, they may enhance the efficacy of SBCE by administering prokinetic agents or by placing the capsule directly into the duodenum with the assistance of an endoscope. The European Society of Gastrointestinal Endoscopy recommends that SBCE may be performed on an outpatient basis whenever possible, as completion rates are generally higher in outpatients than in hospitalized patients (12).

In our study, we found that diabetes is a significant risk factor for DUGT. Previous research has demonstrated that gastrointestinal motility dysfunction occurs in approximately one-third of patients who experience capsule retention, with diabetic gastroparesis being one of the most common disorders contributing to gastrointestinal motility dysfunction in this population (13). A retrospective study conducted by Triantafyllou et al. (14) found that diabetic patients exhibited significantly longer capsule gastric transit times and notably lower rates of complete small bowel examinations compared to non-diabetic patients. Additionally, a prospective European study further corroborated the observation of slower gastric emptying in diabetic patients relative to healthy controls (15). Collectively, these findings indicate that diabetes is a significant risk factor for DUGT. Our study also identified anemia as a risk factor for DUGT in chronic abdominal pain. This association may be attributed to the reduced oxygen supply to the gastrointestinal tract in anemic patients, which can lead to decreased gastrointestinal motility, subsequently resulting in DUGT. Typically, we believe that hypokalemia predisposes individuals to muscular weakness, adversely affecting the motility of gastrointestinal smooth muscle. As potassium deficiency persists, motility throughout the gastrointestinal tract progressively diminishes. In a prospective study conducted by Gan et al. (16) prolonged gastric transit time was observed in patients with hypokalemic compared to those with normal potassium levels; however, this finding was not statistically significant, and hypokalemia was not identified as a risk factor for DUGT in our study. The inconsistency of these findings may be related to the varying levels of potassium reduction and the duration of low potassium levels. Therefore, larger, multicenter, prospective studies are necessary to further evaluate the impact of hypokalemia on SBCE.

A previous retrospective study by Fry et al. (7) demonstrated that the detection rate of SBCE in patients with simple chronic abdominal pain not accompanied by additional symptoms, signs, or abnormal laboratory indices was only 6%. Based on the conclusions of this study, it appeared that the use of SBCE in patients with chronic abdominal pain may have limited value. SBCE is an effective technique, and several subsequent studies have further investigated its value in patients with chronic abdominal pain. These studies actively explored factors that can enhance the detection of positive lesions in this patient population. A prospective multicenter study conducted in Europe (8) involving 50 patients with chronic abdominal pain investigated whether the detection rate was higher in patients with chronic abdominal pain who had concomitant symptoms. The study found that the detection rate of lesions in patients with chronic abdominal pain accompanied by other symptoms or signs was 40%, significantly higher than the rate reported in the study by Fry et al. (7). This indicates that rigorous patient selection based on accompanying symptoms or signs is crucial for enhancing the detection rate of SBCE in patients with chronic abdominal pain. Additionally, inflammation appears to be the most valuable additional sign. Elevated inflammatory markers were also found to be significantly associated with detection rate in patients with chronic abdominal pain in our study. In our study, the lesion detection rate of SBCE in patients with chronic abdominal pain was 30.5%. Among patients with chronic abdominal pain and elevated inflammatory markers, the lesion detection rate increased to 52.3%. It is well established that inflammatory bowel disease, a chronic, non-specific intestinal inflammatory disease, is frequently accompanied by recurrent abdominal pain and abnormal elevations of inflammatory markers in laboratory tests. Consequently, patients with chronic abdominal pain and elevated inflammatory markers are more likely to have lesions in the small intestine detected by SBCE, particularly in cases of inflammatory bowel disease.

In a study of patients with iron deficiency anemia, Nahon et al. (17) concluded that hemoglobin levels below 90 g/L were significantly associated with a higher lesion detection rate during SBCE examinations. A Japanese study involving 578 patients who underwent SBCE further validated the correlation between reduced hemoglobin levels and an increased rate of lesion detection (18). In our study, we found that comorbid anemia in patients with chronic abdominal pain was significantly associated with an elevated lesion detection rate. It is well known that certain chronic hemorrhagic diseases of the gastrointestinal tract, such as malignant tumors, intestinal ulcers, and small intestinal vasodilation, often lead to patients presenting with anemia. However, a German study involving 50 patients with chronic abdominal pain found no significant correlation between anemia and the detection rate of lesions in these patients. We speculate that this discrepancy may be attributed to several factors. First, the definitions of anemia vary, in our study, we employed a stricter definition, classifying anemia as hemoglobin levels below 90 g/L. Second, the gastroenterology department of our hospital tends to admit relatively complex and severe cases, which may influence the detection of lesions to some extent. Third, the sample size of patients with anemia included in this study was relatively small. Therefore, we propose that patients with chronic abdominal pain who also have comorbid anemia should be considered for SBCE when other routine tests yield negative results. Several prior studies have demonstrated that the combination of weight loss or hypoalbuminemia in patients with chronic abdominal pain is significantly associated with a high lesion detection rate. However, in the present study, these two factors, weight loss and hypoalbuminemia, were not examined due to the extensive time span of the data and incomplete recording of certain information.

Our study has several limitations. First, it was a retrospective study that may be subject to selection bias. Second, due to the extended time frame, there were variations in the bowel preparation protocols for the included capsules. And we did not incorporate the bowel scores of the patients into our analysis, which could have influenced the lesion detection rate. Additionally, due to the lack of pathological specimens, the nature of suspected submucosal lesions has not been clearly defined. Finally, we utilized only one brand of capsule and did not consider other brands, such as OMOM, in our study.

In conclusion, our study identified hospitalization status, diabetes, and anemia as significant risk factors for DUGT in patients experiencing chronic abdominal pain. Furthermore, it is essential to closely monitor the capsule running status of these patients in clinical settings. Our findings also highlight the considerable value of SBCE in patients with chronic abdominal pain who present with anemia and elevated inflammatory markers.

## Data Availability

The raw data supporting the conclusions of this article will be made available by the authors, without undue reservation.
